# Mitochondrial DNA in cell death and inflammation

**DOI:** 10.1042/BST20221525

**Published:** 2023-02-23

**Authors:** Rosalie Heilig, Jordan Lee, Stephen W.G. Tait

**Affiliations:** 1 Cancer Research UK Beatson Institute, Switchback Road, Glasgow G61 1BD, U.K.; 2 School of Cancer Sciences, University of Glasgow, Switchback Road, Glasgow G61 1BD, U.K.

**Keywords:** apoptosis, cell death, mitochondria, mtDNA, pyroptosis

## Abstract

Cytosolic DNA is recognized by the innate immune system as a potential threat. During apoptotic cell death, mitochondrial DNA (mtDNA) release activates the DNA sensor cyclic GMP–AMP synthase (cGAS) to promote a pro-inflammatory type I interferon response. Inflammation following mtDNA release during apoptotic cell death can be exploited to engage anti-tumor immunity and represents a potential avenue for cancer therapy. Additionally, various studies have described leakage of mtDNA, independent of cell death, with different underlying cues such as pathogenic infections, changes in mtDNA packaging, mtDNA stress or reduced mitochondrial clearance. The interferon response in these scenarios can be beneficial but also potentially disadvantageous, as suggested by a variety of disease phenotypes. In this review, we discuss mechanisms underlying mtDNA release governed by cell death pathways and summarize release mechanisms independent of cell death. We further highlight the similarities and differences in mtDNA release pathways, outlining gaps in our knowledge and questions for further research. Together, a deeper understanding of how and when mtDNA is released may enable the development of drugs to specifically target or inhibit mtDNA release in different disease settings.

## Introduction

**Figure 1.**
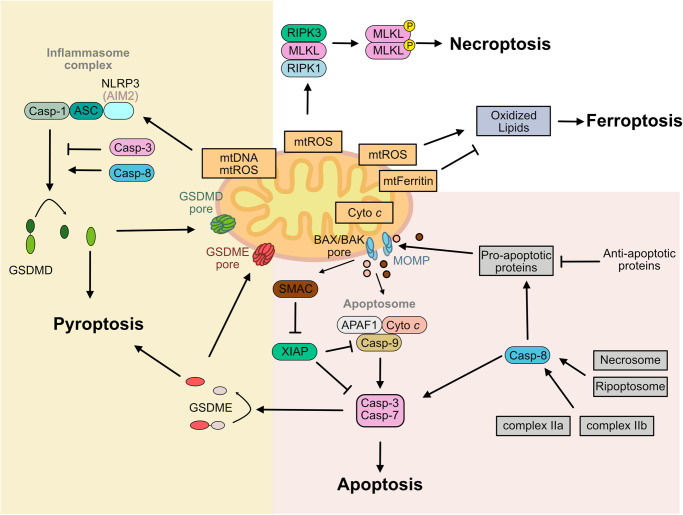
Role of mitochondria in cell death. *Apoptosis*: Mitochondrial apoptosis is held in check by the balance of pro- and anti-apoptotic proteins. An apoptotic trigger promotes BAX BAK oligomerization and lipid pore formation in the mitochondrial outer membrane — mitochondrial outer membrane permeabilisation (MOMP), apoptotosome formation (cyto *c*, APAF1 and caspase-9) in the cytosol and activation of executioner caspases-3 and -7 enforcing cell death. The intermembrane space protein SMAC binds to and inhibits XIAP thereby relieving the brake on caspase-9 and -3 activity. Activation of caspase-8 results in caspase-3 activation and in type II cells, as well as after activation of the ripoptosome and necrosome; apoptotic cell death requires caspase-8 mediated cleavage of BID, mitochondrial outer membrane permeabilization and mitochondrial apoptosis. *Pyroptosis*: mtDNA and mtROS can act as ligands for the inflammasome receptor NLRP3. Activation of the inflammasome platform (receptor, ASC, caspase-1), allows autoproteolytic cleavage and activation of caspase-1, thus promoting cleavage of GSDMD. The N-terminal domain of GSDMD (GSDMD^NT^) inserts into the plasma membrane — promoting pyroptotic cell death. GSDMD^NT^ also forms pores in the mitochondrial membrane. While caspase-3 cleaves GSDMD in its N-terminus (amino acid D87 mouse) deactivating the N-terminal fragment, caspase-8 cleavage of GSDMD results in an active GSDMD^NT^. Activation of caspase-3 can additionally cleave GSDME yielding an active N-terminal fragment promoting pore formation in the mitochondrial and plasma membrane. *Necroptosis*: Mitochondria are not required for the execution of necroptosis, however, mtROS can promote RIP1 kinase phosphorylation and thus promote the initial steps of necroptosis. *Ferroptosis*: Mitochondria impact ferroptosis in at least two ways; firstly, promoting ferroptosis due to mtROS oxidizing mitochondrial lipids and secondly dampening ferroptosis by chelating iron (Abbreviations: MOMP, mitochondrial outer membrane permabilization; APAF1, apoptotic protease-activating factor1; cyto *c*, cytochrome *c*; SMAC, second mitochondria-derived activator of caspases; XIAP, X-linked inhibitor of apoptosis protein; BID, BH3-interacting domain death agonist; mtDNA, mitochondrial DNA; mtROS, mitochondrial reactive oxygen species; NLRP3, NACHT, LRR and PYD domains-containing protein 3; ASC, apoptosis-associated speck-like containing a card; GSDMD, gasdermin-D; RIP1, receptor-interacting protein 1).

Mitochondria are distinct, highly dynamic cellular organelles with myriad functions including, metabolism, energy production, cell death and immune signaling [1,2]. Many of these functions require the double-membrane system compartmentalizing the mitochondria into intermembrane space (IMS) and matrix, separated by an outer and inner mitochondrial membrane (OMM and IMM). Alongside double-membrane compartmentalization, the origin of mitochondria as an archaebacterial endosymbiont also imparts the organelle with its own double stranded, circular genome able to replicate, in the matrix, independently of the nuclear genome [3]. Mitochondria are essential for mitochondrial (intrinsic) apoptosis. Particularly during extrinsic apoptosis [4–8] and intracellular death complexes [9–12] mitochondria can amplify the initial signal leading to cell death. During pyroptosis mitochondria can supply a ligand for inflammasome activation (Figure 1). However, the role of mitochondria in other types of cell death such as necroptosis and ferroptosis is less clear (Figure 1) (see review [13]).

In this review, we focus on signaling pathways specifically induced by the presence of mitochondrial DNA (mtDNA) in the cytosol as a consequence of mitochondrial damage. In addition, attention will be centered on cell death pathways promoting mtDNA release and how this release is mediated.

## Receptors and immune signaling pathways activated by mtDNA

Eukaryotic cells contain a highly elaborate, organellar system ensuring cellular homeostasis. DNA resides in two distinct organelles, nuclei and mitochondria. The presence of DNA in other compartments indicates one of two things: damaged or misfunctioning nucleus and/or mitochondria or the presence of foreign DNA. DNA is detected by DNA sensors in the cytosol or in endocytic vesicles (Toll-like receptor 9 — TLR9) to alert the cell's immune system to misplaced or foreign DNA. The activation of the DNA sensor will govern, in turn, the activation of the signaling pathway and immune response. Two cytosolic pattern recognition receptors and one DNA sensor have specifically been associated with mtDNA detection, the absent in melanoma 2 (AIM2), the NOD-, LRR- and pyrin domain-containing protein 3 (NLRP3) and cyclic GMP–AMP synthase (cGAS).

### mtDNA activates cGAS-STING signaling pathway

**Figure 2.**
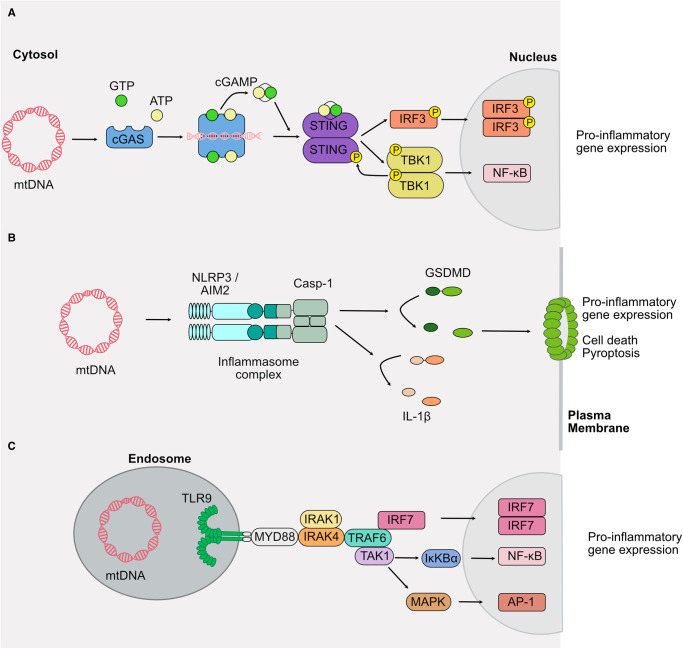
Signaling pathways activated upon mitochondrial DNA recognition in the cytosol. (**a**) Cytosolic mtDNA binds and activates cGAS inducing the production of the second messenger cGAMP and dimerization of STING. STING recruits TBK1 to phosphorylate STING, itself as well as the transcription factor IRF3, thus enabling transcription of pro-inflammatory target genes via NF-κB and IRF3 in the nucleus. (**b**) MtDNA activates the NLRP3 and potentially AIM2 inflammasome receptor which act as a platform for caspase-1 activation and induction of pyroptotic cell death via GSDMD pore formation in the plasma membrane. (**c**) TLR9 recognizes mtDNA in the endosomal compartment and relays the signaling to the adaptor protein MyD88-IRAK1/IRAK4-TRAF6 and IRF7, TAK1-IκKBα or TAK1-MAPK1 which in turn promotes pro-inflammatory gene expression via activation of the transcription factor IRF7, NF-κB or AP-1 (Abbreviations: TLR9, Toll-like receptor 9; mtDNA, mitochondrial DNA; MyD88, myeloid differentiation primary response protein; IRAK, interleukin-1 receptor-associated kinase; TRAF6, TNF receptor-associated factor 6; MAPK, mitogen-activated protein kinase 1; IRF7, interferon regulatory factor 7; cGAMP, cyclic GMP–AMP; cGAS, cyclic GMP–AMP synthase; STING, stimulator of interferon genes protein; IRF3, Inteferon regulatory factor 3; AIM2, absent in melanoma 2; NLRP3, NACHT, LRR and PYD domains-containing protein 3).

cGAS is a DNA-binding protein localized to the cytosol as well as the nucleus [14,15]. While nuclear cGAS activity is tightly held in check, to avoid an unwanted inflammatory response [16–24], binding of dsDNA in the cytosol frees the catalytic site of cGAS, allowing its activation. Active cGAS then synthesizes 2′,3′ GMP–AMP (cGAMP) using ATP and GTP to act as a second messenger activating the endoplasmic reticulum (ER) membrane adaptor STING upon binding [25–28]. The active dimeric STING relocates from the ER to the Golgi compartment, recruits TBK1 which subsequently phosphorylates STING at its C-terminal tail (CTT). Phosphorylated STING acts as a platform for the recruitment and phosphorylation of interferon regulatory factor 3 (IRF3) by TBK1 [29]. Phosphorylated IRF3 (pIRF3) dimerizes and translocates to the nucleus to drive type I interferon transcription (Figure 2). In this review, we specifically highlight the signaling pathways mediated by cytosolic mtDNA promoting cGAS-STING activation resulting in IRF3 but it is important to note that activation of cGAS has also been implicated in the induction of other transcriptional responses mediated by NF-κB [28,30], mitogen-activated protein kinases (MAPK) and STAT factors [31]. Activation of these signaling pathways ensures the transcription of a wide variety of proteins including pro-inflammatory cytokines and type I interferons.

### mtDNA can activate the NLRP3 inflammasome

Activation of the cytosolic pattern recognition receptor NLRP3 leading to inflammasome activation and pyroptosis has been linked to cytosolic mtDNA [32–34]. How the NLRP3 receptor is activated remains unclear, often — but not always — it is linked to sensing potassium efflux [35]. Interestingly, mitochondrial ROS (mtROS) [36,37] as well as leakage of oxidized mtDNA was suggested as an activator of the NLRP3 inflammasome [32–34] (Figure 2). The release of mtROS/mtDNA has been proposed to depend on VDAC (VDAC1 and 2) [36] and the mitochondrial permeability transition pore (mPTP), an unselective (and poorly defined) Ca^2+^ regulated pore in the IMM [32,34,38–40]. How mtROS and mtDNA are detected by the NLRP3 inflammasome remains ambiguous as no DNA recognition/binding domains in the NLRP3 receptor have been identified. One possibility for receptor activation may relate to the leucine-rich repeat domain (LRR) which was proposed to recognize RNA : DNA hybrids during enterohemorrhagic *Escherichia coli* infections [41]. Of note, the bona fide DNA sensor AIM2 can possibly also recognize cytosolic mtDNA [42].

### mtDNA can activate TLR9

In contrast with to cytosolic DNA, the presence of mtDNA in endosomes is detected by a different receptor, TLR9, predominantly expressed in B-cells, plasma cells, Paneth cells and microglial cells [43]. TLR9 drives expression of type I interferons and pro-inflammatory cytokines upon activation of the myeloid differentiation primary response 88 protein (MyD88) which signals through interferon regulatory factor 7 (IRF7), MAPK or NF-kB signaling pathways [44,45]. TLR9 detects specifically unmethylated cytidine-phosphate-guanosine (CpG) dinucleotides, DNA motifs that are frequently found in bacterial DNA (Figure 2) [43,46,47]. The mtDNA can stem various cell types including neutrophils or hepatocytes. Neutrophils release mtDNA due to accumulated oxidized nucleoids or during neutrophil extracellular trap (NET) formation [48–50]. Whereas the mtDNA released by hepatocytes is important during nonalcoholic steatohepatitis (NASH), where the mtDNA is taken up by macrophages inducing inflammation via TLR9 signaling [50–53].

## Cell death pathways can induce mtDNA release

Cell death is important during development, for homeostasis as well as for the clearance of pathogens and destruction of transformed cells. Mitochondria are implicated in many functions ensuring cellular maintenance and survival; thus it is perhaps not surprising that mitochondria and cell death are tightly linked. Interestingly, the activation of two contrasting cell death pathways apoptosis and pyroptosis can result in the release of mtDNA.

### mtDNA release during apoptosis

Apoptosis is divided into extrinsic and intrinsic apoptotic cell death depending on the origin of cell death ligand. Activation of apoptosis from the cell exterior — extrinsic apoptosis — is induced upon binding of cognate ligands to death receptors [4–7], as well as the assembly of intracellular death complexes such as complex IIa, IIb [9,10], ripoptosome or necrosome [11,12] may relay (via tBID) or even require MOMP to drive cell death [8] (Figure 1). Mitochondrial apoptosis is established as an immunologically silent type of cell death [54]. Under homeostasis, apoptosis is held in check by a balance of anti- and pro-apoptotic proteins of the BCL-2 protein family [55]. Upon an apoptotic stimulus such as DNA damage, ER stress, hypoxia or metabolic stress, BH3-only proteins bind pro-survival BCL-2 proteins and pro-apoptotic BAX and BAK proteins thereby activating the latter [56–60]. This enables cytosolic BAX to translocate and embed into the OMM, where BAX and BAK oligomerize to form pores, causing MOMP and release of soluble IMS proteins such as cyto *c* [61–64]. The presence of cyto *c* in the cytosol allows the formation of the apoptosome, a protein complex consisting of APAF1, cyto *c*, inactive caspase-9, enabling activation of caspase-9 and driving apoptotic cell death via caspase-3/-7 activation [13].

**Figure 3.**
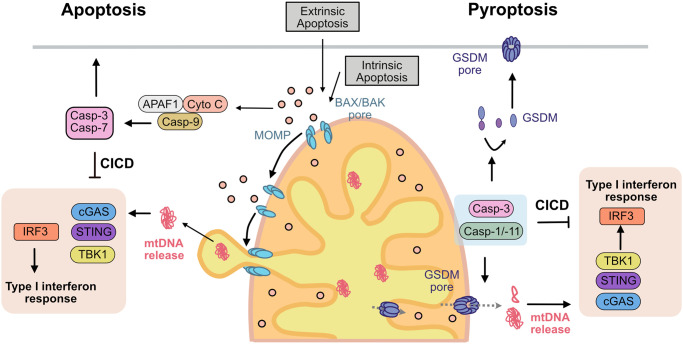
Overview of how mtDNA is released during cell death. *Apoptosis*: Extrinsic or intrinsic apoptosis signals result in BAX/BAK pore formation activation of the apoptotic pathway, extrusion of the inner mitochondrial membrane and release of mtDNA into the cytosol. DNA is recognized by cGAS and the cGAS-STING pathway is activated resulting in IRF3 activation and a type I IFN response. This gene expression is counteracted by caspase-3 cleaving and inactivating cGAS and IRF3. *Pyroptosis*: Activation of gasdermins (GSDMD, GSDME, Gasdermin A3) promotes pore formation in the mitochondria inducing mtDNA release and activation of the cGAS-STING signaling cascade resulting in a type I interferon response. However, the type I IFN reponse is prohibited by caspase-1 and -3 by inactivating cleavage of cGAS and IRF3 (Abbreviations: cGAS, cyclic GMP–AMP synthase; STING, stimulator of interferon genes protein; IRF3, inteferon regulatory factor 3; ISG, interferon stimulatory genes; GSDMD, gasdermin-D; GSDME, gasdermin-E; mtDNA, mitochondrial DNA).

Interestingly, while caspase activity is important during development for cell clearance and cellular organization e.g. in the brain [65,66], APAF1 and caspase-9, -3 and -7 only play a subordinate role compared with BAX/BAK in cellular homeostasis. In particular, APAF1 and caspase-9 deficient cells provide a short-term resistance to apoptotic stimuli, while BAX/BAK deficiency grants full protection [56,67–70]. Similarly, BAX and BAK play a superior role in the clearance of mature blood cell in the hematopoietic stem cell compartment *in vivo* compared with APAF1-, caspase-9 or caspase-3 [71]. These results imply that there are other, caspase-independent, ways for cells to die following MOMP — collectively referred to as caspase-independent cell death (CICD). In contrast with canonical apoptosis, lack of caspases was observed to trigger a type I interferon response prior to death [72–74]. Two independent studies investigated this phenomenon and found that MOMP-induced death in the absence of caspases cause the accumulation of mtDNA in the cytosol activating the cGAS-STING DNA sensing pathway [72,73]. Two separate studies reinforced the previous findings that CICD is pro-inflammatory by visualizing the gradual widening of BAX/BAK macropores allowing the extrusion of the mitochondrial inner membrane, its permeabilization and the release of mtDNA into the cytosol ultimately resulting in transcription of type I interferons (Figure 3) [75,76]. These studies substantiate that apoptosis, a silent type of cell death, can be pro-inflammatory under caspase-deficient conditions. Since the discovery of mitochondrial-driven CICD, additional studies have described a type I interferon response during apoptosis [77,78].

Two consecutive studies have advanced the *in vitro* discovery of CICD by deliberately inducing inflammation using CICD in the tumor microenvironment, thus turning a ‘cold’ tumor ‘hot’ [74,79]. Specifically, activating CICD in a colorectal cancer mouse model prompted a strong anti-tumorigenic immune response driven by infiltration of macrophages and T cells ultimately resulting in regression of the tumor [74]. Subsequent to this, a combination of radiation and the pan-caspase-inhibitor emricasan as a cancer treatment in mice showed synergistic therapeutical effects in cancer regression caused by the inflammatory response of mtDNA released during CICD [79].

The finding that apoptosis can induce inflammation, raises the question of how and why an interferon response is lacking under caspase proficiency, especially since mtDNA is released into the cytosol independent of caspase activity [75] Indeed, caspase-3/7 were shown to cleave essential substrates of the cGAS-STING pathway namely cGAS and IRF3 inactivating these substrates [80]. Additionally, caspases have also been shown to prohibit *de novo* protein translation [81,82]. These findings suggest that caspases harbor a ‘non-cell death function’ important for the regulation of interferon induction by limiting signaling through cGAS-STING and IRF3, as well as protein translation, ultimately prohibiting type I interferon expression (Figure 3) [72,83].

### mtDNA release during pyroptosis

In contrast with canonical apoptosis, pyroptotic cell death is a lytic, highly pro-inflammatory type of cell death. Pyroptosis entails activation of the canonical or non-canonical inflammasome signaling cascade; for canonical signaling this comprises the formation of a multiprotein complex, inflammasome complex, required to activate caspase-1 by autoproteolytic cleavage [84,85]. Activation of the caspase causes cleavage of the cell death substrate gasdermin-D into a N- and a C-terminal domain [86,87]. The N-terminal domain (GSDMD^NT^) translocates to the plasma membrane where it oligomerizes to form large permeability pores promoting cellular lysis [88–91].

Activation of pyroptosis affects mitochondria in different ways: reduction in mitochondrial membrane potential, dysregulating intracellular ion homeostasis, impediment of mitophagy and even permeabilization of mitochondrial membranes [92–95]. As discussed before, mtDNA can act upstream of NLRP3 and possibly AIM2 inflammasome receptor — as a ligand, but inflammasome activation can also cause mtDNA release. It has been shown that the GSDMD^NT^ interacts with lipid species, including cardiolipin, only found in mitochondrial and bacterial membranes, and forms conduits in various lipidic liposomes [88–91]. Besides GSDMD, gasdermin A3 and gasdermin-E (GSDME) have been proposed to target mitochondrial membranes [96,97]. GSDME is cleaved and activated by the apoptotic caspase-3, and pore formation by GSDME promotes cyto *c* efflux [98]. In two recent reports, these findings were extended to show that gasdermin pores (upon activation of the NLRP3 and pyrin inflammasome) can induce mtDNA release directly and independent of cellular lysis (Figure 3) [99,100]. Interestingly, the osmoprotectant glycine was shown to inhibit the release of larger proteins (>25 kDa) as well as mtDNA from GSDM pores in the plasma membrane [101,102], but did not impact release of mtDNA into the cytosol [99].

Notably, similar to apoptotic caspases, the pro-inflammatory caspase-1 can cleave cGAS thereby inhibiting the cGAS-STING pathway (Figure 3) [103]. Moreover, potassium efflux via GSDMD pores was sufficient to impede cGAS-mediated type I interferon activation [95]. These studies suggest that although mtDNA can be released during inflammasome activation, caspase activity as well as changes in cytosolic ion concentrations during pyroptotic cell death may limit the expression of type I interferons.

**Figure 4.**
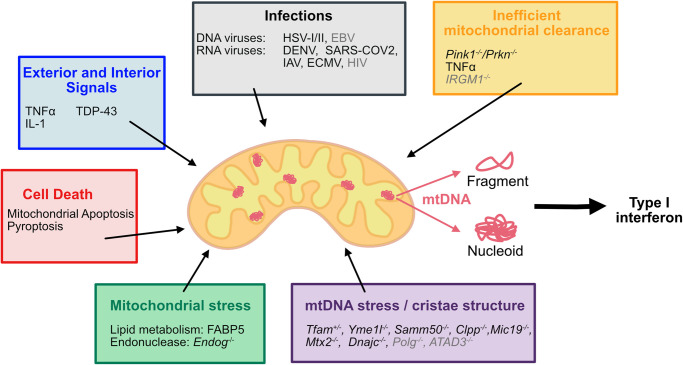
Overview of factors inducing mtDNA release. mtDNA release has been reported in cell death during apoptosis and pyroptosis but also independent of cell death pathways. The non-cell death cues can be categorized in exterior signals, mitochondrial stress, mtDNA stress, inefficient mitochondrial clearance and pathogenic infections. Factors in gray induce a type I IFN response possibly mediating by mtDNA release.

Besides cell death-dependent release of mtDNA many other cell death-independent scenarios have been proposed to also result in mtDNA leakage and cGAS-STING pathway activation. These possibilities are summarized in Table 1 and Figure 4.

**Table 1.** Cell death independent mtDNA release

	Mitochondrial features	Cytosolic DNA	Ref.
**Mitochondrial stress and dysfunction**			
Fatty acid binding protein (FABP5) inhibition in T cells	Distorted cristae structure, altered lipid metabolism, lowered OXPHOS	Cytosolic DNA cGAS-STING activation Type I IFN signature	[104]
*Endog^−/−^*	Accumulation of ROS in MEF cells	Cytosolic DNA cGAS-STING activation Type I IFN signature	[105]
*Tfam^+/−^*	Alteration of mtDNA packaging — bigger nucleoids	Cytosolic DNA cGAS-STING activation Type I IFN signature	[106]
*Ymel^−/−^*	Imbalance of mitochondrial pyrimidine pool	Cytosolic DNA cGAS-STING activation Type I IFN signature	[107]
*ATAD3^−/−^*	Heterozygous mutations in human ATAD3, THP-1 *ATAD3^−/−^*	Cytosolic DNA cGAS-STING activation Type I IFN signature	[108]
*CLPP^−/−^*, *Clpp^−/−^*	Mutation in the protease results in altered nucleoid size and induction of type I interferons	Cytosolic DNA cGAS-STING activation Type I IFN signature	[109]
*Samm50^−/−^*	Inner membrane remodeling induced mtDNA release	Cytosolic DNA cGAS-STING activation Type I IFN signature	[110]
*Mic19^−/−^* *Mtx2^−/−^* *Dnajc^−/−^*	Cristae disorganization induced mtDNA release	Cytosolic DNA cGAS-STING activation Type I IFN signature	[111]
**Reduced and inefficient clearance of mitochondria**			
*Pink1^−/−^/Prkn^−/−^*	Deficiency for PINK or PRK in mice during exhaustive exercise	cGAS-STING activation Type I IFN signature	[112]
TNF-α	Down-regulation of PINK1 expression, decreased autophagy	Cytosolic DNA	[113,114]
*IRGM1^−/−^*	Accumulation of defective mitochondria — higher mtROS, lower respiratory capacity, more depolarized mitochondria	Indirect effect	[115]
**Infections**			
HSV-I/II — herpes simplex I/II	Degradation of TFAM and mtDNA by viral nucelase	Type I IFN signature	[106]
SARS-COV2 — s*evere acute respiratory syndrome coronavirus 2*	Virus induced mtDNA release in endothelial cells	Cytosolic DNA cGAS-STING activation Type I IFN signature	[116]
DENV — *dengue virus*	NSB2 a DENV protease prohibits cGAS detection of mtDNA	Cytosolic DNA cGAS-STING activation Type I IFN signature	[117,118]
IAV — *influenza A virus* ECMV — *encephalomyocarditis virus*	Viroporins	Cytosolic DNA	[119]
**Extracellular cues inducing mtDNA release**			
IL-1β	In myeloid, fibroblast and epithelial cells	Cytosolic DNA cGAS-STING activation Type I IFN signature	[120]
**Intracellular cues inducing mtDNA release**			
TDP-43	Cytoplasmic accumulation of the nuclear DNA/RNA binding protein TDP-43 induces mtDNA release	Cytosolic DNA cGAS-STING activation Type I IFN signature	[121]

## Mechanisms of mtDNA release

Many studies have documented escape of mtDNA into the cytosol, but how is a large, negatively charged DNA nucleoid that resides in the mitochondrial matrix detected by a cytosolic DNA sensor cGAS? Does the DNA pass through two lipidic membranes into the cytosol? Are membranes permeabilized to facilitate its release?

### BAX/BAK macropores

During apoptosis, the release of mtDNA into the cytosol is dependent on pro-apoptotic proteins BAX and BAK, thus suggesting the requirement of BAX/BAK pores to perforate the OMM. BAX/BAK form dynamic pores in the OMM upon oligomerization [122–126]. The inner membrane then protrudes through the BAX/BAK macropore and must thus be large enough to allow the passage of mtDNA [75,76]. Considering that cGAS could not be detected inside the protruding inner membrane [75,76]; breaching of the inner membrane likely facilitates mtDNA detection by cGAS. The exact mechanism of mitochondrial inner membrane permeabilization remains unclear. However, several possibilities are excluded; for example, mitochondrial dynamics (fusion and fission) does not impact mtDNA release [76]. In addition, rupture of the inner membrane due to the prolonged opening of the mPTP and ensuing IMM swelling could also be excluded on the basis that lack of mPTP regulator cyclophilin D did not reduce mtDNA release [76]. Nevertheless, the data does not exclude that permeabilization of the IMM is a passive event. New data however indicate shedding of large portions of the outer membrane prompted by *Toxoplasma gondii* infection was not sufficient to breach the IMM [127]. This may indicate that permeabilization of the IMM requires more than disruption of the OMM.

### Gasdermin pores

Activation of gasdermin A3, D and E during pyroptosis have been suggested to trigger mtDNA release. Since the N-terminal domain of gasdermin forms large pores in membranes and has been shown to release soluble cytosolic proteins during pyroptosis [101,102,128], it was perhaps natural to assume that mtDNA can be released via gasdermin pores. The gasdermin pore however would have to perforate both mitochondrial membranes, IMM and OMM or allow extrusion of the inner membrane when the outer membrane is perforated. The outer diameter of the GSDM pore was measured to 28/40 nm (GSDMA3/GSDMD) and the inner diameter between 10 and 18 nm [88–91,129]. Although gasdermin pores clearly interact with cardiolipin and other lipids, the measured pore size is too small to transfer full-sized (80 × 100 nm) nucleoids. Thus, two possibilities for mtDNA release are probable, release as a result of collateral damage or a direct release of mtDNA fragments over the pore.

### Mitochondrial permeability transition pores and VDAC oligomers

An early report proposed the release of mtDNA fragments via mPTP [130], which was described to have a minimal diameter of 2.8 nm [130–132]. A more recent report shows mtDNA release from *Endog*^−/−^ cells being independent of apoptosis. Instead, *Tfam*^+/−^ and ENDOG-deficient cells release mtDNA fragments into the IMS upon Ca^2+^ and ROS-induced mPTP opening [105]. In a second step the mtDNA fragments are released upon VDAC1 (possibly VDAC3) oligomerization — not part of the mPTP — from the outer mitochondrial membrane [105]. Since the release of mtDNA observed in cells deficient for YME1L or TDP-43 invasion into mitochondria, is independent of BAX/BAK pores but requires VDAC oligomerization it is likely also released via mPTP and VDAC oligomers [107,121]. Molecules smaller than 1.5 nm have been suggested to permeate the mPTP pore suggesting nucleoids are too big, but mtDNA fragments (DNA molecules in solution 0.33 nm [133]) can pass through the IMM into the IMS [134]. According to the NMR structure of human VDAC1, the pore has an approximate 3.4 nm diameter, and would thus be large enough to allow passage of mtDNA fragments (PDB 6TIQ) [135]. It was further suggested that mtDNA interacts with the VDAC1 to stabilize the pore; however, what signals promote dissociation to allow release of mtDNA through the pore remains ambiguous. Interestingly, double-stranded mtDNA (dsmtDNA) breaks are immediately cleared rather than repaired [136,137]. Thus, it is puzzling, that fragments of dsmtDNA are not degraded but instead released through mPTP and VDAC1 pores. Possibly this is suggestive of a protective mechanism to inhibit mtDNA degradation.

## Open questions and future perspectives

The detection and reaction of the cell to mtDNA in the cytosol is multifold. It is dependent on cell type and availability of receptor, but also on the way mtDNA is released (apoptotic conditions, etc.). This complicates the study of how mtDNA is released and activates cGAS-STING signaling. Many questions such as how and if mtDNA is fragmented before release, how mtDNA exits the IMM, how cGAS gets access to mtDNA and recognizes mtDNA, and whether the characteristics of mtDNA play a role in its recognition (circularity, CpG methylation, proteins and packaging of mtDNA) remain to be answered. It is also unclear how much mtDNA is required for the activation of cGAS, whether longer DNA stretches induce a more potent inflammatory response as suggested previously [138], and whether mtDNA leakage can occur in absence of mitochondrial rupture. In particular, can limited mitochondrial permeabilization — minority MOMP cause DNA damage and genome instability in absence of cell death [139], but can it also be a source of inflammation as suggested for a variety of pathogenic infections [140]? Furthermore, it is unclear whether mtDNA recognition by cGAS can be masked and potentially regulated, as reported for nuclear cGAS interacting with histones, thus limiting its activation by the nuclear genome [16,22–24]. Does mitochondrial TFAM adopt a similar role? Future research should address some of these questions, as elucidation and characterization of mtDNA is of importance to further our understanding in the role of mtDNA in health and disease as well as exploit the pathway pharmacologically.

Inflammation induced by the release of mtDNA is double-sided and can be beneficial as well as detrimental for the cell and organism. For instance, the release of mtDNA by BAX/BAK pores, in absence of caspases, induces pro-inflammatory cGAS-STING and NF-κB signaling which is important to repress tumorigenesis and induce tumor clearance. In addition, inhibition of the autophagic machinery fueling mtDNA release can potentially have synergistic therapeutical effects in combination with radiation in breast cancer cells [74,77]. Treatment of cancers using a combination therapy of apoptosis inducers and caspase inhibitors could thus improve therapeutic outcome. Activation of cGAS-STING pathway and expression of type I interferons has also shown to be beneficial in various virus infections to counteract viral proliferation and limiting viral spread [141–143].

Mitochondrial DNA release can also be unfavorable, for example promoting disease progression in systemic lupus erythematosus (SLE), amyotrophic lateral sclerosis (ALS), rheumatoid arthritis (RA) and others [105,114,121]. It is seen that inhibition of VDAC pores, thereby inhibiting mtDNA release, reduces disease severity in a mouse model of SLE [105]. Moreover, the hallmark protein TDP-43 in ALS was shown to induce mtDNA release via mPTP, linking mtDNA release to the ALS disease phenotype [121]. Blocking of mtDNA release in this setting would thus ameliorate the disease phenotype.

Understanding and elucidating the mechanism of mtDNA is of high importance pharmacologically. Hopefully, it will be employed to develop future treatment for cancer in respect to limiting cancer growth. But also used to develop agents hindering mtDNA release to limit progression in diseases phenotypes associated to mtDNA release.

## Perspectives

- *Importance in the field.* Escape of mtDNA into the cytosol is associated with the up-regulation of an inflammatory, type I interferon-driven response. Understanding the cues and factors inducing leakage of mtDNA into the cytosol in cell death and beyond is of importance to purposely promote (e.g. in cancer, infections) or hinder (mtDNA-associated disease phenotypes) mtDNA release pharmacologically.
- *Summary of the current thinking.* mtDNA is released upon MOMP during apoptosis in caspase-deficient conditions. During apoptosis the cytosolic mtDNA activates the DNA sensor cGAS inducing a NFκB and type I interferon response, thus promoting inflammation. Besides apoptosis, cues such as mitochondrial, stress, mtDNA stress, pathogenic infections and others have been found to induce mtDNA release by distinct mechanisms causing inflammation via activation of the DNA sensors (cGAS) and receptors (NLRP3, AIM2 or TLR9).
- *A comment on future directions*. To date, mtDNA release has been observed in a variety of systems, circumstances and reasons resulting in inflammatory gene expression. However, mechanistical insight into how mtDNA reaches the cytosol as well as how recognition is regulated is still ambiguous and will require further efforts. Furthermore, the impact of mtDNA-driven inflammation in health and disease is of high importance for pharmacological intervention and will require in-depth knowledge of the pathway.

## Abbreviations

AIM2: absent in melanoma 2
ALS: amyotrophic lateral sclerosis
cGAS: cyclic GMP–AMP synthase
CICD: caspase-independent cell death
CpG: cytidine-phosphate-guanosine
ER: endoplasmic reticulum
IMM: inner mitochondrial membrane
IMS: intermembrane space
IRF3: interferon regulatory factor 3
IRF7: interferon regulatory factor 7
LRR: leucine-rich repeat
MAPK: mitogen-activated protein kinases
mPTP: mitochondrial permeability transition pore
mtDNA: mitochondrial DNA
OMM: outer mitochondrial membrane
SLE: systemic lupus erythematosus
